# Verrucous carcinoma of buccal mucosa in female - a rare case scenario

**DOI:** 10.11604/pamj.2022.41.141.32024

**Published:** 2022-02-17

**Authors:** Nishanth Gurunathan, Krishna Prasanth Baalann

**Affiliations:** 1 Department of Oral Pathology and Microbiology, Sree Balaji Dental College and Hospital, Bharath Institute of Higher Education and Research, Chennai, Tamil Nadu, India,; 2 Department of Community Medicine, Sree Balaji Medical College and Hospital, Bharath Institute of Higher Education and Research, Chennai, Tamil Nadu, India

**Keywords:** Verrucous carcinoma, Ackerman’s tumour, parakeratin plugging

## Image in medicine

Verrucous carcinoma is a warty variant of squamous cell carcinoma. It most often affects the oral cavity, with the buccal mucosa being the most common site. It is more prevalent among tobacco users with male predominance. Here, that scenario changed. A 42-year-old female patient presented with a chief complaint of discomfort in the left side of her mouth that had been present for 7 days. History revealed patient had a habit of chewing quid for 7-8 times each day. On general examination, the patient exhibited a normal walk and posture, as well as being well oriented, aware, and moderately built. On intraoral examination, a proliferative verrucous growth was seen across the left buccal mucosa, extending anteroposteriorly from the retrocommissural region to the posterior buccal mucosa and superoinferiorly from the upper buccal vestibule to about 1 cm above the lower vestibule. The lesion was 7x4 cm in size, clearly defined, and had irregular borders. The surface was uneven at the periphery, with finger-like projections in the centre. The colour ranged from reddish in the peripheral region to white in the centre. On palpatory findings, size, location, surface, and form were validated. With uneven and firm borders, the lesion was painful and raised from surrounding mucosa. Incisional biopsy of the lesion was done. Hematoxylin and eosin (H and E) section showed stratified squamous epithelium with hyperplastic in nature and down growth into the cellular connective tissue. Parakeratin plugging was found in a number of cleft-like areas. The basal cell layer showed hyperplasia and some cells with mitotic activity were noticed. Infiltration of many darkly stained inflammatory cells was seen in the underlying connective tissue. Final diagnosis of verrucous carcinoma was given. For treatment, surgical excision was done and advised for regular follow-up.

**Figure 1:**
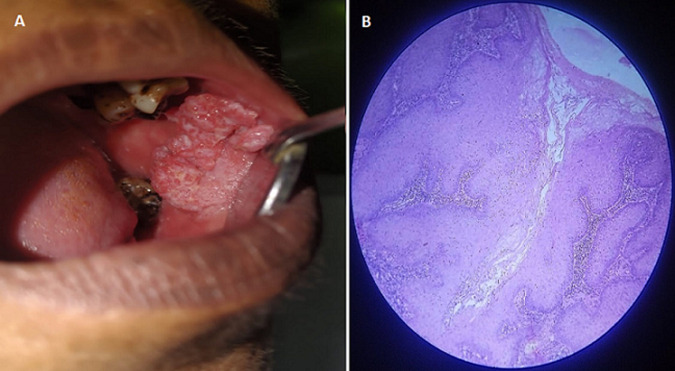
A) proliferative growth in buccal mucosa; B) hyperplastic epithelium with parakeratin plugging seen, numerous inflammatory cells infiltration also noticed

